# Thermal conductivity enhancement in gold decorated graphene nanosheets in ethylene glycol based nanofluid

**DOI:** 10.1038/s41598-020-71740-1

**Published:** 2020-09-07

**Authors:** M. C. Mbambo, M. J. Madito, T. Khamliche, C. B. Mtshali, Z. M. Khumalo, I. G. Madiba, B. M. Mothudi, M. Maaza

**Affiliations:** 1 UNESCO-UNISA Africa Chair in Nanosciences-Nanotechnology, College of Graduate Studies, University of South Africa, Muckleneuk Ridge, PO Box 392, Pretoria, South Africa; 2 iThemba LABS-National Research Foundation, 1 Old Faure Road, PO Box 722, Somerset West, Western Cape Province 7129 South Africa; 3 Department of Physics, College of Science, Engineering and Technology, University of South Africa, Private Bag X6, Florida, Johannesburg, 1710 South Africa

**Keywords:** Energy science and technology, Materials science, Nanoscience and technology

## Abstract

We report on the synthesis and thermal conductivity of gold nanoparticles (AuNPs) decorated graphene nanosheets (GNs) based nanofluids. The GNs-AuNPs nanocomposites were synthesised using a nanosecond pulsed Nd:YAG laser (wavelength = 1,064 nm) to ablate graphite target followed by Au in ethylene glycol (EG) base fluid to obtain GNs-AuNPs/EG hybrid nanofluid. The characterization of the as-synthesised GNs-AuNPs/EG hybrid nanofluid confirmed a sheet-like structure of GNs decorated with crystalline AuNPs with an average particle diameter of 6.3 nm. Moreover, the AuNPs appear smaller in the presence of GNs which shows the advantage of ablating AuNPs in GNs/EG. The thermal conductivity analysis in the temperature range 25–45 °C showed that GNs-AuNPs/EG hybrid nanofluid exhibits an enhanced thermal conductivity of 0.41 W/mK compared to GNs/EG (0.35 W/mK) and AuNPs/EG (0.39 W/mK) nanofluids, and EG base fluid (0.33 W/mK). GNs-AuNPs/EG hybrid nanofluid displays superior enhancement in thermal conductivity of up to 26% and this is due to the synergistic effect between AuNPs and graphene sheets which have inherent high thermal conductivities. GNs-AgNPs/EG hybrid nanofluid has the potential to impact on enhanced heat transfer technological applications. Also, this work presents a green synthesis method to produce graphene-metal nanocomposites for various applications.

## Introduction

The enhancement of the efficiency of heat transfer fluids has been of great interest in the cooling industry for emerging advanced technologies. Conventional heat transfer fluids such as oil, water, ethylene glycol possess poor heat conduction properties^1,2^. For instance, the thermal conductivity of these fluids is lower than 1 W/mK while that of metals and their corresponding oxides are 2 to 3 magnitudes higher^3^. Thermal conductivity of heat transfer fluids is critical in determining the efficiency of an engine, due to this problem the numerical methods have been adopted to improve the thermal conductivity of heat transfer fluids. Recently, nanofluids have been proposed as potential heat transfer fluids^1,2,4–6^. For instance, Hou et al. have shown that nanofluids transfer heat much faster than water, indicating that the fabricated Boron nitride nanosheets nanofluids have excellent thermal transfer property^7^. Nevertheless, the enhancement of the thermal conductivity of nanofluids depends on various factors like particle size, temperature, volume fraction, pH, viscosity, etc.

Carbon nanotubes and graphene-based nanofluids become the most promising enhanced heat transfer fluids due to their high thermal conductivity and relatively low mass density than other nanoparticles like copper, aluminium oxide (Al_2_O_2_) and titanium dioxide (TiO_2_)^8–10^. Amiri et al. prepared crumpled nitrogen-doped graphene nanosheet based water-ethylene glycol coolant which exhibited a high thermal conductivity compared to that of water-EG base fluid^11^. Yu et al. prepared stable nanofluids by dispersing graphene oxide nanosheets in ethylene glycol and reported a high thermal conductivity for nanofluids as compared to the EG based fluid^12^. Choi et al. prepared multiwalled carbon nanotubes (MWCNTs) in oil suspensions by a two-step method: (1) MWCNTs were produced in chemical vapour deposition reactor and thereafter, (2) were dispersed into a synthetic poly (α-olefin) oil^8^. Their work showed an enhancement in thermal conductivity for carbon nanotubes based nanofluids. Yang Fu et al. synthesised nanocomposites of graphene oxide (GO) and gold nanoparticles (AuNPs) for generating solar steam under sunlight irradiation^13^. It was found that the efficiency of solar vapor generation of GO-Au nanofluids increased by 10.8% with only 15.6 wt.% of Au nanoparticles which indicated the enhancement of steam generation efficiency^13^.

Moreover, the thermal conductivity of carbon nanotubes and graphene were shown to improve by decorating their surfaces with metal nanoparticles^14^. Torres-Mendieta et al. demonstrated an effective way to produce graphene metal assemblies by femtosecond radiation-based technique through which they anchored AuNPs onto the surface of graphene oxide sheets inside deionized (DI) water by Laser radiation of a gold disk surrounded by graphene oxide suspension^15^. Yarmand et al. decorated functionalized graphene nanoplatelets with Ag nanoparticles inside DI water by chemical synthesise route^16^. From this nanofluid, the experimental data showed improvements in thermal conductivity and heat transfer efficiency in comparison with the corresponding base-fluid^16^. Jha et al. reported an enhancement in thermal and electrical conductivities of nanofluids based on MWCNTs decorated by Ag, Au, and Pd metal nanoparticles using a chemical reduction method with DI water and EG as base fluids^14^. Their work showed a maximum enhancement of 37.3% and 11.3% in thermal conductivity for Ag-MWCNTs nanofluid with DI water and EG as base fluids, respectively, at a volume fraction of 0.03%.

The synthesis methods of graphene-metal nanoparticles hybrid nanofluids are a new and interesting research topic for enhanced heat transfer technological applications^14–18^. Herein, we report on a nanosecond pulsed Nd:YAG laser synthesis of graphene nanosheets decorated with Au nanoparticles in ethylene glycol to obtain GNs-AuNPs/EG hybrid nanofluid. Among the high conductivity (both heat and electricity) metallic nano-sized particles, AuNPs are explored for various nanotechnology-related applications, considering thus their nontoxicity and unique optical, physicochemical and biological properties^19^. These particles can be synthesised with an average particle size of ~ 5 nm or less, and reproducibly. The readily-accessible surface of the AuNPs with a highly increased stability allows the particles to be physically-embedded within the surface of GNs. The characterization of the as-synthesised GNs-AuNPs/EG hybrid nanofluid confirmed a sheet-like structure of GNs decorated with crystalline AuNPs with an average particle diameter of 6.3 nm. The thermal conductivity analysis displayed high thermal conductivity for the as-synthesised GNs-AuNPs/EG hybrid nanofluid compared to GNs/EG and AuNPs/EG nanofluids, and EG base fluid.

## Experimental

### Materials and synthesis

**Figure 1:**
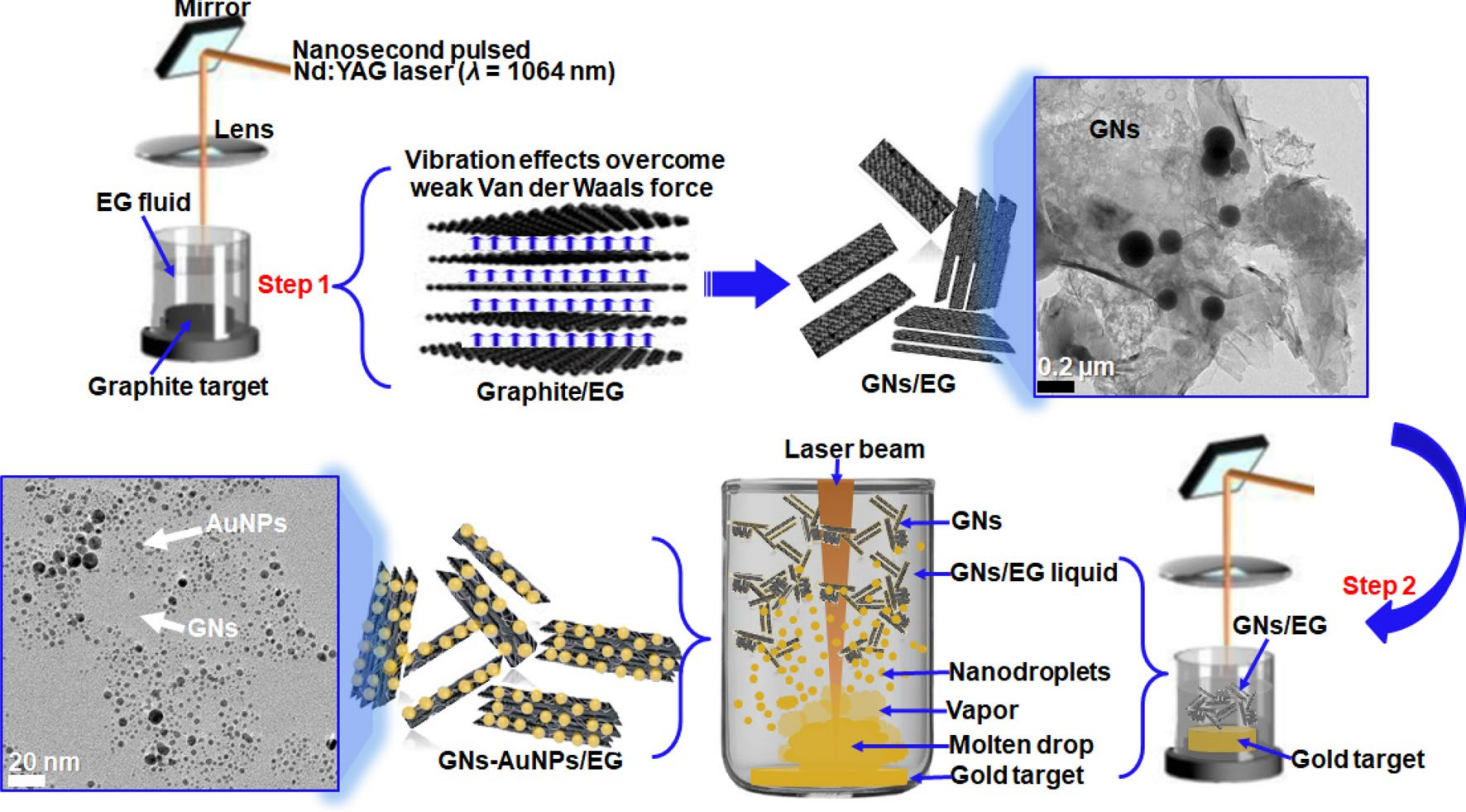
The schematic illustration of the synthesis route of GNs-AuNPs/EG hybrid nanofluid by pulsed Nd:YAG laser ablation of a graphite and Au targets in EG and GNs/EG media, respectively.

Graphite and gold targets with purity of 99.99%, and ethylene glycol with purity of 99.8% were purchased from Sigma Aldrich. The synthesis route of GNs-AuNPs/EG hybrid nanofluid by pulsed Nd:YAG laser ablation of a graphite and gold targets in EG and GNs/EG media, respectively, is presented in Fig. 1 by a schematic illustration. From this figure, two experimental parts/steps were performed: In step 1, a 3 mm thick graphite target with 10 mm diameter was placed at the bottom of a glass-beaker filled with 20 mL of EG. Thereafter, a nanosecond pulsed Nd:YAG laser with a wavelength of 1,064 nm, pulse duration of 6–7 ns, repetition rate of 10 Hz and output energy of 85 mJ/pulse was used to produce GNs in EG with the processing time of 5–30 min. The pulsed laser beam was focused through a convex lens with a focal length of 300 mm on a graphite target. In nanosecond pulsed Nd:YAG laser ablation, the thermal wave has enough time to propagate into the target material and ablate it. In the graphite target (step 1), there is an induced vibration of graphite layers due to mismatch between the acoustic impedances of graphite and EG which causes tensile stress between graphitic layers^20^. For vibration effects that overcome the weak Van der Waals force between graphitic layers, graphite is exfoliated into few-layer graphene sheets (GNs) which are dispersed in EG^20–22^. In step 2, the graphite target at the bottom of a glass-beaker now filled with GNs/EG liquid was replaced by a 1 mm thick gold target with 5 mm diameter. The gold ablation was carried out for 15–20 min. In this case, when a laser beam heats the metal target, plasma, vapor, and metal micro- or nanosized droplets are generated as initial products, which further react with the liquid medium to form nanoparticles anchored on GNs^23^. In Fig. 1, the micrograph of GNs/EG nanofluid shows a sheet-like structure of GNs and that of GNs-AuNPs/EG hybrid nanofluid displays AuNPs dispersed on the surface of GNs. The average lateral size of GNs flakes is ~ 0.5 to 1 µm.

### Characterization

The morphological characterisation of the samples was carried out using a high-resolution transmission electron microscopy (HR-TEM), JEOL JEAM 2010F microscopy equipped with LaB_6_ filament and operated at 200 kV. The energy dispersive X-ray (EDX) in HR-TEM was used for elemental composition analysis. The scanning electron microscopy (SEM) images were obtained using high-resolution scanning electron microscope (HRSEM, Leo-Stereo-Scan of 40) operated at 5.0 kV. The X-ray diffraction (XRD) was carried out using SmartLab (Rigaku) diffractometer with a scanning rate of 0.2° s^−1^ and 2θ range 20°–90°, operating with a Cu tube (*λ* = 0.15406 nm) at 50 kV and 30 mA. WITec alpha 300 confocal Raman microscope with 532 nm excitation laser was used to characterize the as-synthesized samples. Raman spectra were measured at room temperature with the laser power of 4 mW. Fourier transform infrared (FT-IR) spectroscopy analysis was performed in the range of 500 to 4,000 cm^−1^ using Perkin Elmer ATR spectrometer. Thermal conductivity of the as-synthesised nanofluids was carried out by a simplified transient hot-wire technique (known as Guarded hot plate (GHP) method).

The as-synthesised nanofluids were transferred into centrifuge tubes for sonication at 3,000 rpm for 10 min, thereafter, the suspensions of each tube were collected and dried and weighed to obtain the particles concentrations. Furthermore, a mixture of as-synthesised GNs-AuNPs/EG hybrid nanofluid was sonicated in the ultra-sonication bath for an hour to attain a uniform and homogeneous dispersal of GNs-AuNPs in the solution. Thereafter, 5 ml of this solution was mixed with 5 ml of EG base fluid and sonicated for 15 min, afterwards, the solution was collected for thermal conductivity analysis. This procedure was repeated for GNs/EG and AuNPs/EG nanofluids. Briefly, GHP method is recognised as the absolute method for thermal conductivity measurement in the steady-state of materials and can achieve a global measurement uncertainty below 2%^24^. The main principle of this method is to reproduce the uniform, unidirectional and constant thermal flux density existing through a sample fixed between two infinite isothermal planes, and the method operates within the temperature-controlled limits of 25 to 45 °C. Thermal conductivity was calculated using a one-dimensional Fourier Eq. ^24^.

1 $$k = \frac{\phi d}{{A\left( {T_{h} - T_{c} } \right)}}$$

where *ϕ* (W) is the heat flow-rate that in an ideal unidirectional condition would traverse the specimen through an area, *A* (m^2^), called measurement area. The variable *d* (m) is the thickness of the nanofluid between the hot and cold plates, whereas the thermal flux (*ϕ*) is equal to the electric power applied to the heater. The percentage enhancement in thermal conductivity was calculated using the relation

2 $$\% = \frac{{\left( {K_{n} - K_{f} } \right) \times 100}}{{K_{f} }}$$

where *K*_*f*_ and *K*_*n*_ is the thermal conductivity of the base fluid and nanofluid, respectively.

## Results and discussion

**Figure 2:**
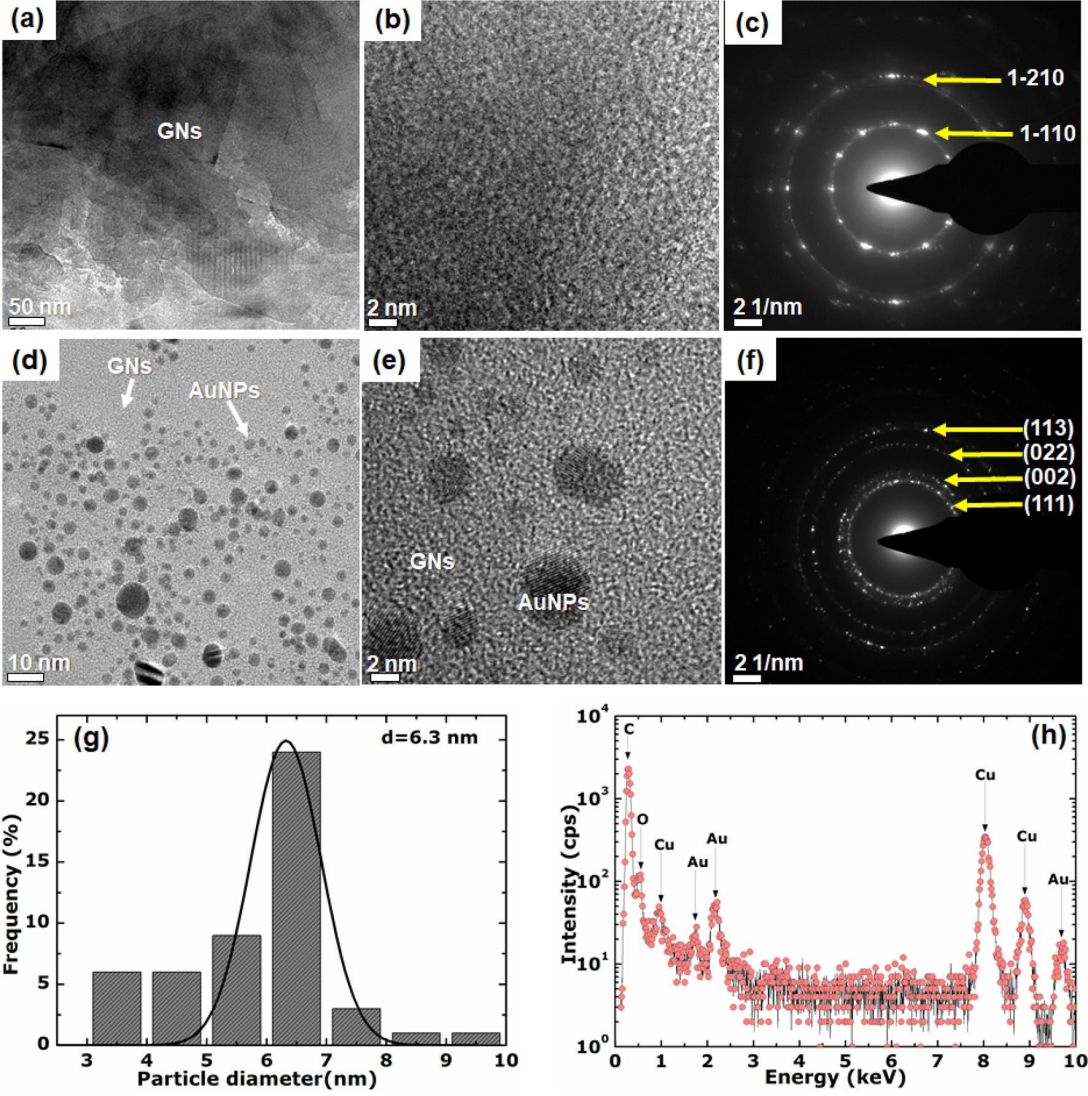
(**a**,**b**) Low and high resolution TEM micrographs of GNs of as-synthesised GNs/EG nanofluid, respectively, and the corresponding (**c**) SAED pattern of GNs. (**d**,**e**) Low and high resolution TEM images of AuNPs anchored on GNs of as-synthesised GNs-AgNPs/EG hybrid nanofluid, respectively, and the corresponding (**f**) SAED pattern of AuNPs. (**g**) AgNPs size distribution histogram (with Gaussian distribution curve) for AuNPs in GNs-AgNPs/EG hybrid nanofluid. (**h**) EDX spectrum of GNs-AuNPs/EG hybrid nanofluid.

**Figure 3:**
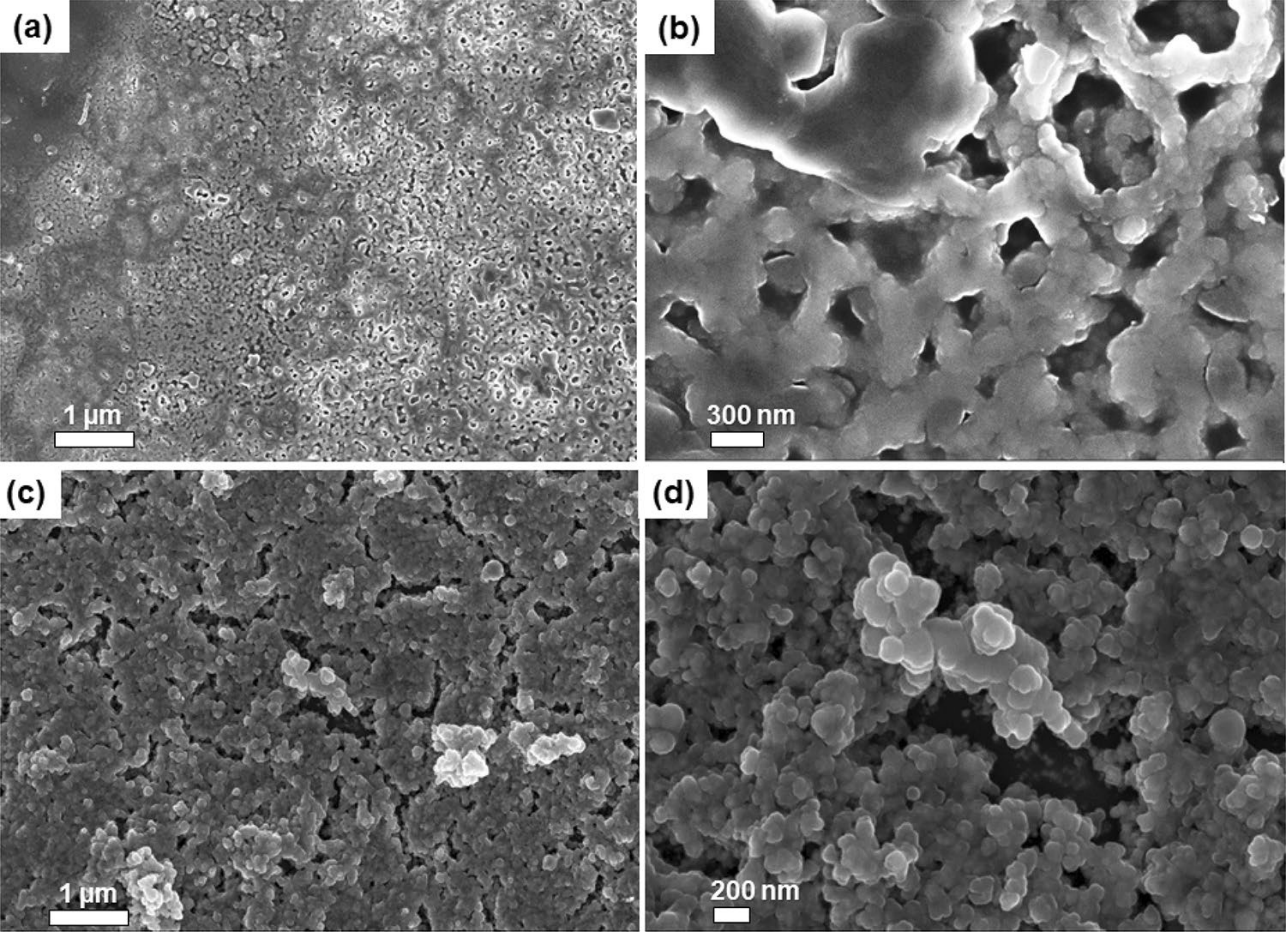
(**a**,**b**) Low and high magnification SEM micrographs of as-synthesised GNs-AuNPs/EG hybrid nanofluid, respectively. (**c**,**d**) Low and high magnification SEM images of as-synthesised AuNPs in EG without GNs.

Figure 2 and 3 presents the HR-TEM and SEM images, respectively, of the as-synthesised GNs/EG and GNs-AuNPs/EG nanofluids. In Fig. 2a, b, the as-synthesised GNs/EG nanofluid shows a typical sheet-like structure of a few-layer graphene sheets with lateral size of ~ 0.5 to 1 µm which shows a selected area electron diffraction (SAED) pattern (Fig. 2c) corresponding to that of a few-layer graphene. The SAED pattern of GNs displays an interlayer spacing (*d*-spacing) of approximately 0.123 nm corresponding to Miller-Bravais indices (1–210) for outer ring and *d* = 0.213 nm corresponding to (1-110) indices for the inner ring^25^. On the other hand, in Fig. 2d, e, GNs-AuNPs/EG hybrid nanofluid shows spherical crystalline AuNPs anchored on the surface of GNs confirming a successful synthesis of the hybrid nanofluid. The SAED pattern of AuNPs anchored on GNs (Fig. 2f) displays diffraction rings corresponding to (111), (002), (022) and (113) planes of gold which confirms the crystallinity of the nanoparticles. The SAED pattern of AuNPs (Fig. 2f) was indexed to a face-centered cubic (FCC) structure of Au with a space group of *Fm-3m* using the best matching Inorganic Crystal Structure Database (ICSD) card #64701 for Au. Furthermore, Fig. 2g presents AuNPs size distribution histogram with Gaussian distribution which shows that the particle size is mainly within the range of 3–10 nm, with an average particle diameter of 6.3 nm. Furthermore, to evaluate the elemental composition of the GNs-AuNPs/EG hybrid nanofluid the EDX analysis was carried out, as shown in Fig. 2h. This figure shows the main elements (C and Au) of the hybrid nanofluid composition which confirms the presence of graphene decorated with AuNPs. The presence of Cu originates from the TEM grid and the observed oxygen could be due to the presence of oxygen-containing groups of GNs in a hybrid nanofluid.

Figure 3a,b and Figure 3c,d present the SEM images of the as-synthesised GNs-AuNPs/EG hybrid nanofluid and AuNPs/EG nanofluid (i.e. without GNs), respectively. It is worth mentioning that for comparison purposes, AuNPs were also synthesised in EG without GNs. The distribution of AuNPs anchored on the surface of GNs can be seen from the low magnification SEM image of as-synthesised GNs-AuNPs/EG hybrid nanofluid. The observed bright spots could be attributed to the formation of metal nanoparticles on the surface of graphene^26^. The SEM images of both GNs-AuNPs/EG and AuNPs/EG nanofluids shows Au particles agglomeration. However, it can be seen that AuNPs appear smaller in GNs-AuNPs/EG nanofluid as compared to AuNPs/EG nanofluid which shows the advantage of ablating AuNPs in GNs/EG.

**Figure 4:**
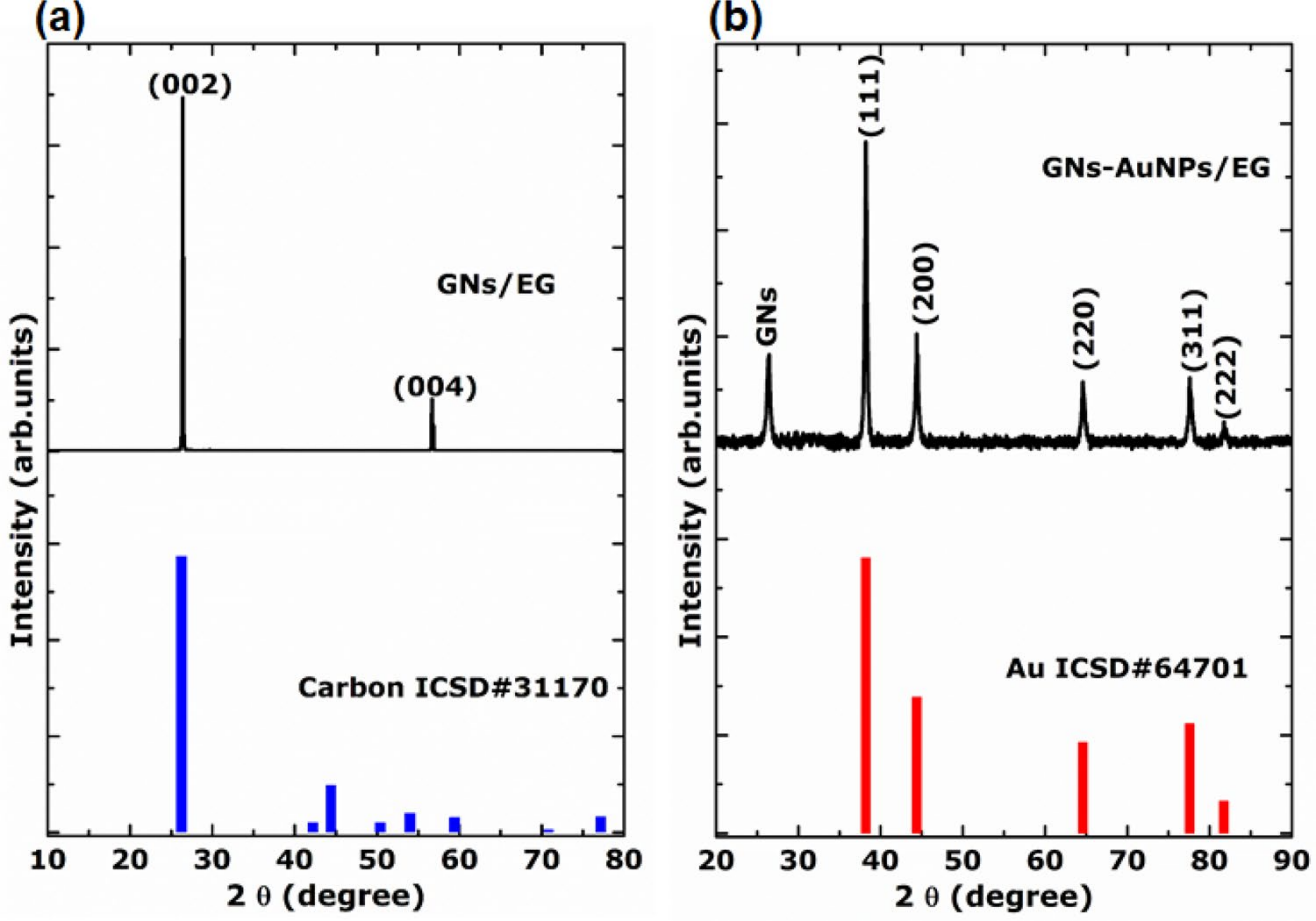
XRD patterns: (**a**) GNs of as-synthesised GNs/EG nanofluid and the corresponding carbon ICSD card. (**b**) AuNPs anchored on GNs of as-synthesised GNs-AuNPs/EG hybrid nanofluid and the corresponding gold ICSD card.

Figure 4a,b show the XRD patterns of the as-synthesised GNs/EG and GNs-AuNPs/EG nanofluids, respectively. The XRD pattern of the as-synthesised GNs/EG nanofluid (Fig. 4a) displays the typical diffraction peaks of a few-layer graphene sheets which were indexed using the carbon ICSD card #31170. An intense (002) diffraction peak at 2*θ* = 26.7°, corresponding to interplanar spacing of 0.343 nm confirms a successful synthesis of GNs. The (004) peak in GNs/EG nanofluid does not correspond well to the ICSD card and this could be due to the lattice strain present in the natural graphite flake resulting from the laser interaction with graphite and the presence of the functional groups. In Fig. 4b, it can be seen that the AuNPs anchored on GNs of as-synthesised GNs-AuNPs/EG hybrid nanofluid are crystalline and the diffraction peaks were indexed using the gold ICSD card #64701 of the cubic Au crystal with a space group *Fm-3m*.

**Figure 5:**
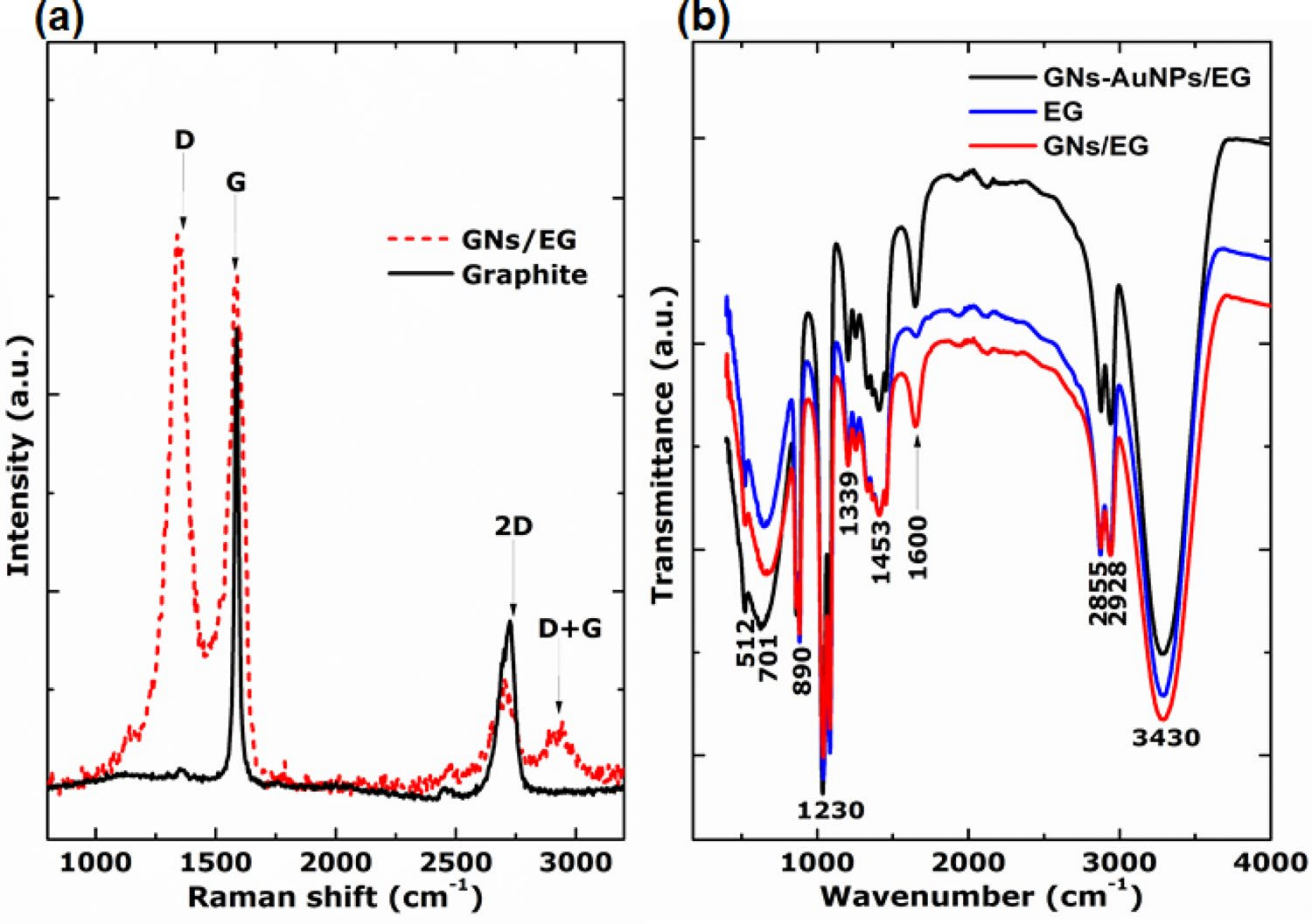
(**a**) Raman spectra of GNs of as-synthesised GNs/EG nanofluid and graphite target. (**b**) FTIR spectra of the as-synthesised GNs-AuNPs/EG and GNs/EG nanofluids and that of the EG base fluid.

The chemical structures of the as-synthesized nanofluids were further investigated using the Raman and FTIR vibration spectroscopy. The Raman spectra show the G band (~ 1,602 cm^−1^) which involves in-plane bond stretching displacements of sp^2^ carbon atoms (E_2g_ symmetry) and 2D band (~ 2,670 cm^−1^) which originates from the second-order process (double resonance Raman process) that involves two in-plane transverse optical mode (iTO) phonons near the *K*-point^27,28^. In Fig. 5a, the Raman spectrum of graphite target shows characteristic of an undisturbed graphitic structure (only shows the G (~ 1,602 cm^−1^) and 2D (~ 2,670 cm^−1^) bands). The G band originates from the in-plane bond stretching displacements of sp^2^ carbon atoms (E_2g_ symmetry) and 2D band from the second-order process (double resonance Raman process) that involves two in-plane transverse optical mode (iTO) phonons near the *K*-point^27,28^. Furthermore, the Raman spectrum of the GNs (Fig. 5a) of the as-synthesised GNs/EG nanofluid displays an additional first-order band (D band at ~ 1,350 cm^−1^), which is a breathing mode of sixfold rings (A_1g_ symmetry) and is known to characterize disorder in the graphitic structure. This is a typical Raman spectrum of few-layer graphene synthesised from graphite target and shows a minimum ratio of the disordered carbon relative to the graphitic carbon (D/G) of about 1.2.

Figure 5b shows the FTIR spectra of the as-synthesised GNs-AuNPs/EG and GNs/EG nanofluids and that of the EG base fluid. These spectra show a broad peak at 3,430 cm^−1^ which is due to the hydroxyl (O–H) group on the 2-dimensional plane of the carbon skeleton and the peak located at 1,728 cm^−1^ is assigned to the C=O stretching vibration of the carboxyl groups^29,30^. The peaks located at 2,928 and 2,855 cm^−1^ are assigned to C-H, while the peak located at 1,600 cm^−1^ is assigned to C=C bonds associated with the GNs carbon skeleton structure^29,30^. Moreover, the peaks located at 1,453, 1,379, 890 and 701 cm^−1^ are attributed to C–O (epoxy and alkoxy) bonds^31^. There is a noticeable increase in the intensity of the 1,600 cm^−1^ peak of C=C bonds for nanofluids with GNs as compared to EG base fluid which further confirms a successful synthesis of graphene. The spectra also show absorption peaks at 512 cm^−1^ which arise from hydroxyl groups of EG chains^32^, and a slight difference in the intensities of these peaks for nanofluids suggest that there is a bonding of C and Au with oxygen from hydroxyl groups of EG chains. Also, the FTIR spectrum of EG is the same as that of the as-synthesised nanofluids which confirms that during synthesis the chemical structure of the base fluid was not altered by laser.

Moreover, the nanofluids solution prepared for thermal conductivity analysis, i.e. 5 ml of as-synthesised AuNPs/EG and GNs-AuNPs/EG mixed with 5 ml of EG base fluid and sonicated for 15 min, was analysed with EDX to obtain Au concentration as show in Table 1. The EDX spectra obtained are similar to the spectrum in Fig. 2h with the presence of Cu originating from the TEM grid. It is worth noting that in both samples the gold ablation was carried out under the same experimental conditions hence they yield the equivalent amounts of Au.

**Table 1:** EDX fractional concentrations of elemental compositions of as-synthesised AuNPs/EG and GNs-AuNPs/EG nanofluids.

Sample	C (wt.%)	O (wt.%)	Au (wt.%)	Cu (wt.%)
AuNPs/EG	76.34	12.60	1.03	10.03
GNs-AuNPs/EG	78.82	14.74	0.72	5.72

**Figure 6:**
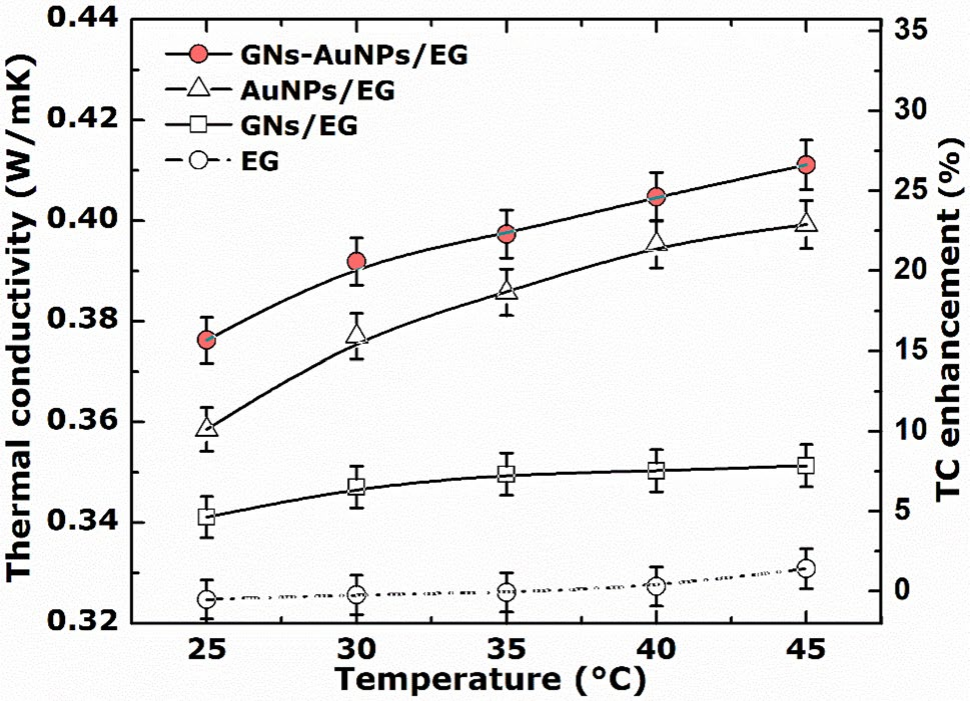
Thermal conductivity (TC) and the corresponding enhancement of the as-synthesised GNs-AuNPs/EG, GNs/EG and AuNPs/EG nanofluids, and EG base fluid as a function of the temperature.

The thermal conductivity of the nanofluids was measured as a function of temperature with uncertainty below 2%, as shown in Fig. 6. From this figure, the thermal conductivity of the as-synthesised GNs-AuNPs/EG nanofluid is compared to that of GNs/EG, AuNPs/EG nanofluids, and EG base fluid. As seen in Fig. 6, the thermal conductivity of the nanofluids is increasing nonlinearly with temperature, and the linearity/nonlinearity of thermal conductivity with temperature and/or volume fraction depends on the nature of the nanoparticle as well as the base fluid. The thermal conductivity of the base fluid and GNs/EG nanofluid show a slight increase of ~ 1.8% and 3%, respectively with increasing temperature in the range of 25–45 °C. EG base fluid inherently possess poor heat transfer characteristics^1,2^, and an increase in its thermal conductivity with temperature in the presence of GNs (i.e., GNs/EG nanofluid) could be attributed to the decrease in interfacial thermal resistance between base fluid and solid nanoparticles at high temperatures^33^, and high thermal conductivity of graphene. Similarly, in the presence of metal nanoparticles, i.e., AuNPs/EG and GNs-AuNPs/EG nanofluid the enhanced thermal conductivity which shows a significant increase with temperature is due to the synergistic effect between AuNPs and graphene which have inherent high thermal conductivities. In addition, such change of thermal conductivity with temperature has been thought to be due to Brownian motion of nanoparticles^34–37^. It is worth noting that the thermal conductivity of the nanofluid would also increase with volume fraction. However, in our work we are not reporting the effect of volume fraction on thermal conductivity of the nanofluid since in our experiment the nanoparticles were directly synthesised inside the base fluid.

Moreover, from thermal conductivity measurements, it can be seen that GNs-AuNPs/EG hybrid nanofluid exhibits enhanced thermal conductivity of 0.41 W/mK compared to GNs/EG (0.35 W/mK) and AuNPs/EG (0.39 W/mK) nanofluids, and EG base fluid (0.33 W/mK). GNs/EG nanofluid displays superior thermal conductivity enhancement compared to some of the previously reported thermal conductivity enhancement of graphene-based ethylene glycol nanofluids, as shown in Table 2. Also, GNs-AuNPs/EG hybrid nanofluid displays superior enhancement in thermal conductivity of up to 26% in the temperature range 25–45 °C (Fig. 6), which is higher compared to other previously reported thermal conductivity enhancement of graphene nanocomposites-based ethylene glycol nanofluids (Table 3).

**Table 2:** A comparison of thermal conductivity enhancement of graphene-based ethylene glycol nanofluids reported in the literature from various preparation methods and those reported in this work.

Preparation method	Particle type	Base fluid	Particle concentration	Measurement technique	Maximum enhancement	Temperature (°C)	References
Pulsed Nd:YAG laser ablation	Graphene nanosheets (GNs)	Ethylene Glycol (EG)	0.06 wt.%	Guarded hot plate	8%	25–45	This work
Modified Hummers method	Exfoliated graphene	EG	0.005–0.056 vol.%	Transient hot-wire (THW) method	4–7%	25–50	^17^
Hydrogen exfoliated graphene (HEG)	Exfoliated graphene	EG	0.05–0.08 wt.%	THW method	1–5%	25–50	^38^
Modified Hummers method	Alkaline graphite oxide	EG	0.008–0.138 vol.%	THW method	2.4–6.5%	25	^39^
Multiwalled carbon nanotubes (MWNTs) prepared by chemical vapour deposition	MWNTs	EG	0.04%	KD2 pro thermometer	7.3%	28–50	^14^

**Table 3:** A comparison of thermal conductivity enhancement of graphene nanocomposites-based ethylene glycol nanofluids reported in the literature from various preparation methods and those reported in this work.

Preparation method	Particle type	Base fluid	Particle concentration	Measurement technique	Maximum enhancement	Temperature (°C)	References
Pulsed Nd:YAG laser ablation	Gold nanoparticles (AuNPs)	EG	0.08 wt.%	Guarded hot plate	22%	25–45	This work
Pulsed Nd:YAG laser ablation	Graphene nanosheets /AuNPs	EG	0.12 wt.%	Guarded hot plate	26%	25–45	This work
Chemical reduction method	Gold nanoparticles coated with Multiwalled nanotubes (MWNTs)	EG	0.03%	KD2 pro thermometer	9.7%	28–50	^14^
Modified Hummers method to prepare the GO	Silver nanoparticles decorated graphene	EG	0.01–0.07 wt.%	THW method	3–14%	25–70	^40^
Chemical reduction method	Palladium nanoparticles coated with MWNTs	EG	0.03%	KD2 pro thermometer	8.5%	28–50	^14^
Chemical reduction method	Copper oxide decorated graphene (CuO/HEG)	EG	0.01–0.07 wt.%	THW method	17–23%	25–50	^41^

**Figure 7:**
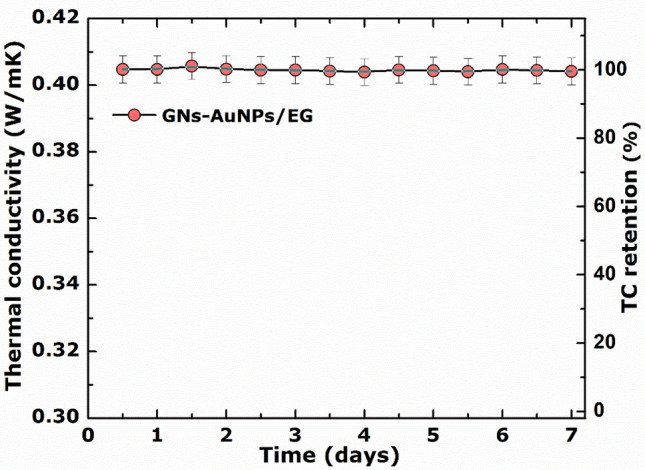
Thermal conductivity (TC) retention as function of time for the as-synthesised GNs-AuNPs/EG nanofluids at a temperature of 40 °C.

Moreover, the suspension stability of the as-synthesised GNs-AuNPs/EG nanofluid was tested for 7 days at 40 °C without any visible particle sedimentation. GNs-AuNPs/EG nanofluid showed excellent thermal conductivity stability with retention of ~ 100% at a temperature of 40 °C over 7 days, as shown in Fig. 7.

## Conclusion

We have successfully synthesised graphene nanosheets decorated with Au nanoparticles (average particle diameter = 6.3 nm) in ethylene glycol by ablating graphite target followed by Au in ethylene glycol base fluid using a nanosecond pulsed Nd:YAG laser (wavelength = 1,064 nm) to obtain GNs-AuNPs/EG hybrid nanofluid. The characterization of the as-synthesised GNs-AuNPs/EG hybrid nanofluid confirmed a sheet-like structure of GNs decorated with crystalline AuNPs. The thermal conductivity measurements showed that GNs-AuNPs/EG hybrid nanofluid exhibits enhanced thermal conductivity of 0.41 W/mK as compared to the EG base fluid (0.33 W/mK). GNs-AuNPs/EG hybrid nanofluid displays superior enhancement in thermal conductivity of up to 26% in the temperature range 25–45 °C, which is higher compared to other previously reported thermal conductivity enhancement of EG based nanofluids. The high thermal conductivity of GNs-AgNPs/EG hybrid nanofluid is due to the synergistic effect between AuNPs and graphene which have inherent high thermal conductivities. On the other hand, the low thermal conductivity of the base fluid is due to the high viscosity of EG^38^. These results make this hybrid nanofluid suitable for enhanced heat transfer technological applications.

It is worth mentioning that the effect of particle loading on heat transfer and the viscosity of nanofluids has been extensively studied by number of researchers and showed that heat transfer increased by increasing the volume concentration of nanoparticles. In our work, we are not reporting the effect of volume concentration of nanoparticles on heat transfer enhancement since in our experiment the nanoparticles were directly synthesised inside the base fluid. However, in the future we will investigate the viscosity of the as-synthesised nanofluids under different experimental conditions.
